# Developing a Modified Low-Density Lipoprotein (M-LDL-C) Friedewald's Equation as a Substitute for Direct LDL-C Measure in a Ghanaian Population: A Comparative Study

**DOI:** 10.1155/2018/7078409

**Published:** 2018-12-31

**Authors:** Richard K. D. Ephraim, Emmanuel Acheampong, Swithin M. Swaray, Enoch Odame Anto, Hope Agbodzakey, Prince Adoba, Bright Oppong Afranie, Emmanuella Nsenbah Batu, Patrick Adu, Linda Ahenkorah Fondjo, Samuel Asamoah Sakyi, Beatrice Amoah

**Affiliations:** ^1^Department of Medical Laboratory Science, School of Allied Health Sciences, University of Cape Coast, Ghana; ^2^Department of Molecular Medicine, School of Medical Sciences, Kwame Nkrumah University of Science and Technology, Ghana; ^3^School of Medical and Health Sciences, Edith Cowan University, Western Australia, Australia; ^4^Department of Biochemistry, Dalian Medical University, China

## Abstract

Despite the availability of several homogenous LDL-C assays, calculated Friedewald's LDL-C equation remains the widely used formula in clinical practice. Several novel formulas developed in different populations have been reported to outperform the Friedewald formula. This study validated the existing LDL-C formulas and derived a modified LDL-C formula specific to a Ghanaian population. In this comparative study, we recruited 1518 participants, derived a new modified Friedewald's LDL-C (M-LDL-C) equation, evaluated LDL-C by Friedewald's formula (F-LDL-C), Martin's formula (N-LDL-C), Anandaraja's formula (A-LDL-C), and compared them to direct measurement of LDL-C (D-LDL-C). The mean D-LDL-C (2.47±0.71 mmol/L) was significantly lower compared to F-LDL-C (2.76±1.05 mmol/L), N-LDL-C (2.74±1.04 mmol/L), A-LDL-C (2.99±1.02 mmol/L), and M-LDL-C (2.97±1.08 mmol/L) p < 0.001. There was a significantly positive correlation between D-LDL-C and A-LDL-C (r=0.658, p<0.0001), N-LDL-C (r=0.693, p<0.0001), and M-LDL-C (r=0.693, p<0.0001). M-LDL-c yielded a better diagnostic performance [(area under the curve (AUC)=0.81; sensitivity (SE) (60%) and specificity (SP) (88%)] followed by N-LDL-C [(AUC=0.81; SE (63%) and SP (85%)], F-LDL-C [(AUC=0.80; SE (63%) and SP (84%)], and A-LDL-C (AUC=0.77; SE (68%) and SP (78%)] using D-LDL-C as gold standard. Bland–Altman plots showed a definite agreement between means and differences of D-LDL-C and the calculated formulas with 95% of values lying within ±0.50 SD limits. The modified LDL-C (M-LDL-C) formula derived by this study yielded a better diagnostic accuracy compared to A-LDL-C and F-LDL-C equations and thus could serve as a substitute for D-LDL-C and F-LDL-C equations in the Ghanaian population.

## 1. Background

Cardiovascular diseases (CVDs) are the leading cause of morbidity and mortality globally [1, 2]. High LDL-C is of longstanding clinical and research interest as it is an independent risk factor for cardiovascular events and coronary heart diseases (CHDs) [3, 4]. The advent of lipoprotein cholesterol measurement led to epidemiologic analyses that considered the potential effects of the various particles on cardiovascular (CVD) risk [5]. Previous studies have clearly incriminated high levels of LDL-C as atherogenic and have established a link between elevated LDL-C and cardiovascular events [6, 7].

Since the inception of Friedewald's LDL-C equation in 1972, it has been used most widely to estimate LDL-C in clinical practice as well as in health screenings [8]. The American National Cholesterol Education Program (NCEP) working group on lipoprotein measurements recommended that LDL-C concentration be determined with a total analytical error not exceeding ±12% (≤4% imprecision and ≤4% inaccuracy) to guarantee correct patient classification into NCEP risk categories [8]. However there have been numerous attempts to improve its accuracy and reliability in population-based studies [9–12]. Regardless of the importance of accurate evaluation for LDL-C, the Friedewald's formula with its inherent remarkable deviation and limitation continues to be used in clinical and research settings as a cost-effective method to estimate LDL-C when triglyceride levels are less than 4.52 mmol/l [13].

Ultracentrifugation and beta-quantitation are the gold standards for LDL-C measurement [14]. Other methods include direct measurement of LDL-C using a homogenous assay. These methods are expensive, inconvenient, and not readily available in most routine laboratories. Not only is ultracentrifugation as a separation method tedious and time-consuming, but high salt concentrations and centrifugal forces can substantially alter high labile lipoproteins [15, 16]. Furthermore, the diagnostic performance of direct measurement of LDL-C is limited by high triglycerides (TG) levels [17]. In addition, the concentrations of TGs in the various lipoprotein fractions are known to be heterogeneous and therefore change with lipid disorders and other conditions [17, 18].

Previous studies have shown that the formula underrates LDL-C and CV risk stratification even when triglyceride levels are below 4.52 mmol/l [13, 19]. Following the aforementioned drawbacks, Anandaraja et al. and Vojovic et al. have attempted to derive formulas that are specific for Indian and Serbian population, respectively [11, 20]. Martin et al. also provided an alternative to improve LDL-C estimation in a South African population but proposed that external validation and further modifications were needed to improve its utilization [9].

The heterogeneity of population, as well as differences in dietary habits, calls for a more population-specific LDL-C formula that will be generic, accurate, and precise. The scarcity of literature on modified LDL-C formula in a West African population makes it necessary that we begin to document and validate existing formulas in our setting. Research in this direction will provide the breakthrough in combating the burden of atherosclerosis and serve as a useful guide for stakeholders in the management and control of cardiovascular diseases in Ghana. Using fasting lipid profile data from patients who visited the laboratory department of the National Cardiothoracic Centre, this study validated the existing LDL-C formulas and derived a modified LDL-C formula specific to a Ghanaian population.

## 2. Materials and Methods

### 2.1. Study Design/Site

This was a comparative study for the estimation of LDL-C using three different formulas and direct estimation by a homogenous assay. Data was collected for the lipid profile samples received in the laboratory unit of the National Cardiothoracic Centre (NCC) in Accra from December 2016 to April 2017. The NCC in Korle Bu, Accra, is one of the few functioning referral centres in West Africa where complete evaluation of cardiothoracic diseases is not only possible but very safe and of a standard comparable internationally.

### 2.2. Ethical Consideration

This study was approved by the Committee on Human Research, Publication and Ethics (CHRPE) of School of Medical Sciences, KNUST. The subjects were adequately informed of the purpose, procedures, nature, risks, and minimal discomfort of the study. Participants were coded and assured of strict anonymity, confidentiality, and the freedom to exit or decline participation at any time without penalty.

### 2.3. Sample Size and Selection of Participants

Samples for lipid profile analysis were collected from patients visiting the laboratory unit of the NCC over the period stated. This was after participants had given their consent. Of a total of 1540 samples analysed, 22 were excluded because they had triglyceride levels greater than 4.52mmol/l (400mg/dl). The sample size of 1518 (N=1518) was comprised of 782 males and 736 females.

### 2.4. Inclusion and Exclusion Criteria

Participants with no evidence of metabolic conditions (diabetes, chronic renal failure) as per clinical history and had observed at least ten (10) hours of overnight fasting were included. Samples with triglyceride levels greater than 4.52mmol/l (400mg/dl) were excluded.

### 2.5. Sample Collection and Biochemical Assays

A volume of at least 3 mL of venous blood was taken into plain tubes after a 12-hour or minimum of 8-hour overnight fast via phlebotomy. The blood could clot, and serum was separated by centrifugation (2000g for 10mins) and analysed on URIT 8210 automatic chemistry analyser by TC and TG were measured enzymatically by CHOD-PAP and Glycerol phosphate peroxidase-PAP methods, respectively, using reagent kit obtained from Human Diagnostic Worldwide, Germany. TC and TG were calibrated using general chemistry calibrator provided by Human Diagnostic. The reagent kit for direct LDL-C assay, the Chema LDL direct FL test, was manufactured by Hospitex Diagnostics, Italy. HDL-c measurement was performed using a direct homogenous method without precipitation with the use of enzymatic colorimetric assay provided by Human Diagnostic, Germany. LDL-C concentration was directly determined by enzymatic assays.

LDL-C concentrations were also calculated by Friedewald's, Anandaraja's and Martin's formula as follows:

 
*F-LDL-C = TC – HDL – (TG/5)*
 
*A-LDL-C = (0.9TC) – (0.9XTG/5) – 28*
 
*N-LDL-C = TC – HDL-C – TG/Novel Factor* (all calculated in mg/dL).

### 2.6. Derivation of M-LDL-c in a Ghanaian Population

Re-examination of Friedewald's formula for LDL-C determination in our setting is based on the current results, following the procedure which led to Friedewald's formula derivation. Factor for VLDL-C concentration estimation was recalculated. Total cholesterol, TG, LD-C, and HDL-C concentration measurements were used in the initial group to calculate the VLDL-C/TG ratio for a Ghanaian population. The sum of HDL-C and LDL-C was subtracted from TC for each person. This accounted for the assessment of VLDL-C concentration for each person. Afterwards, to determine the mean of the ratio, the TG concentration was divided by the corresponding calculated VLDL-C. The mean ratio, TG/ VLDL, was 4 compared to 2.2 according to Friedewald, M-LDL-C (mmol/L) =TC-HDL-C-TG/4.0 [11]

### 2.7. Data Analysis

Data collected were stored in MS Excel spread sheet. Using the Statistical Package for Social Sciences program (SPSS, version 21.0 for Windows) and GraphPad prism (Version 5 for windows, Inc. 2007), statistical analyses were carried out. Results are expressed as means ± SD and percentages in parenthesis. Unpaired t-test and one-way ANOVA were used to compare mean values of continuous variables for two and more than two categories. Person's correlation analysis was used to determine the association between directly measured LDL-C and calculated LDL-C. The Bland-Altman plots for comparison were used to determine level of bias and agreement of the calculated LDL-C to direct LDL-C. Linear regression analysis was used to generate linear models for the estimation of LDL-C. For all statistical comparisons, a* P* < 0.05 was considered as statistically significant.

## 3. Results

Lipoprotein concentrations and their distributions in the validation group are given in Table 1. The D-LDL-C values were significantly lower than F-LDL-C, ALDL-C, N-ALDL-C, and M-ALDL-C values (p < 0.001). The mean absolute bias amongst calculated LDL-Cs compared to the direct method was 0.29 ± 0.34 mmol/L for Friedewald's formula, 0.27 ± 0.33 mmol/L for Martin's formula, 0.57 ± 0.31 mmol/L for Anandaraja's formula, and 0.50 ± 0.34 mmol/L for modified formula.

The mean %ΔLDL between calculated LDL-Cs compared to the direct method was 12.39 ± 27.34% for Friedewald's formula, 11.74 ± 29.25% for Martin's formula, 23.54 ± 30.45% for Anandaraja's formula, and 21.33± 28.43% for modified formula (Table 2).

The ability of the formulas to correctly classify subjects at the clinical decision cut-off points in specific subgroups is shown in Table 3. Separate analysis was done for subgroups defined by cut-off values (ranges) for TC, TG, and D-LDL-C values provided by NCEP ATPIII guidelines. There were significantly lower mean values of F LDLC in the first quartile of TC compared to D-LDLC and higher mean values in the rest of the quartiles for TC, and few ranges of TG and of LDL-C. A-LDL-C were significantly higher (p < 0.001) than most ranges of TC in the D-LDL-C. A-LDL-C showed no significant difference compared to D-LDL-C in TG and LDL-C. N-LDL-C was significantly lower in the first range of TC in the D-LDL-C but significantly higher in the rest of the ranges of TC in D-LDL-C (p < 0.001). N-LDL-C showed no significant difference compared to D-LDL-C in TG and LDL-C except 3.10±0.93 and 2.31±0.65, respectively (p < 0.001). M-LDL-C was significantly higher (p < 0.001) compared to D-LDLC in all ranges of TC and in most ranges of TG and of LDL-C (Table 3).

From Table 4, a cut-off value of 2.92 mmol/L F-LDL-C had a sensitivity of 0.63 and specificity of 0.84 with negative predictive value of 0.87 and positive predictive 0.74. With N-LDL-C, cut-off value 2.92 was used; this yielded a sensitivity of 0.63 and specificity of 0.85 for detection of LDL-C with positive predictive value of 0.78 and negative predictive value of 0.87. A-LDL-C had sensitivity of 0.68 and specificity of 0.73. M-LDL-C had a sensitivity of 0.60 and specificity of 0.88 with a cut-off value of 3.23mmol/L.

**Figure 1:**
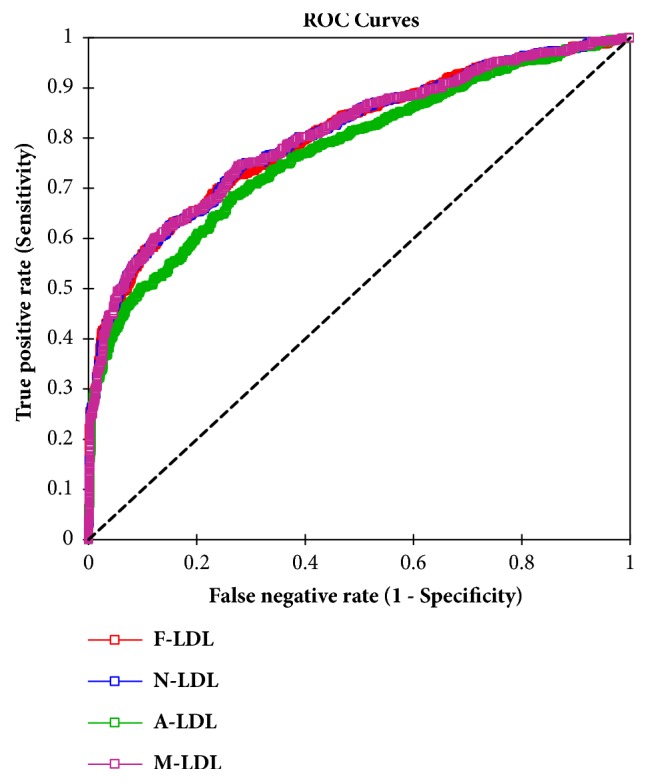
ROC curves depicting the accuracy of the different forms of LDL-C measurements.

Figure 1 shows receiver operating characteristics (ROC) curve analyses for depicting the accuracy of Friedewald's formula (F-LDL-C), Martin's formula (N-LDL-C), and Anandaraja's formula (A-LDL-C) and M-LDL-C, LDL-C calculated by modified formula. Area under curve (AUC) was 0.80 for F-LDL-C, 0.81 for N-LDL-C, 0.77 for A-LDL-C, and 0.81 for M-LDL-C.

Correlation analysis between various formulas is used to estimate the LDL-C concentrations. With respect to the Pearson correlation coefficient (r) and coefficient of determination (R^2^), various formulas were strongly positive correlated with each other (p<0.0001) (Table 5).

**Figure 2:**
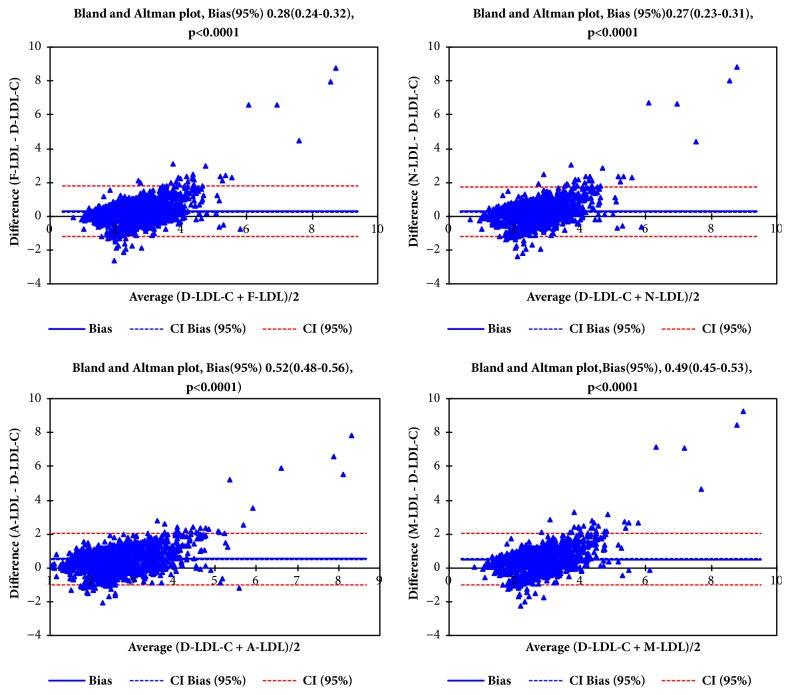
Bland and Altman plot of the different forms of LDL-C measurements (F-LDL-C, N-LDL-C, A-LDL-C, and M-LDL-C).

Figure 2 shows Bland-Altman plots for direct LDL-C against Friedewald's formula (F-LDL-C), Martin's formula (N-LDL-C), and Anandaraja's formula (A-LDL-C) and M-LDL-C, LDL-C calculated by modified formula. The mean bias for F-LDL-C was 0.28(0.24-0.32), 0.27(0.23-90.31) for N-LDL-C, 0.52(0.48-0.56) for A-LDL-C, and 0.49(0.45-0.53) for M-LDL-C.

## 4. Discussion

Currently, the NCEP guidelines focus on diagnosis and treatment of TC and LDL-C. It is therefore relevant to accurately estimate LDL-C, as it has significant implications on cardiovascular risk stratification and can affect therapy and outcomes. The gold standard methods for quantifying LDL-borne cholesterol in serum are laborious and thus poorly suited to the modern laboratory [21]. Furthermore, many kinds of equipment and tubes are used, making conditions difficult to reproduce from one laboratory to another and consistent separations highly dependent on the skills and care of the technician [16]. Nevertheless, ultracentrifugation remains the classic comparison method and is the basis for the accepted reference methods [16, 17, 21]. Numerous studies have been conducted to derive more precise formulas for LDL-C calculations in the past decades in different populations compared to the globally used Friedewald's formula [11, 20–22]. However, some of these modifications were not found to be suitable replacements of the Friedewald formula. This present study sought to compare the diagnostic performances of the Friedewald's (F-LDL-C), Martin's (N-LDL-C), and Anandaraja's (A-LDL-C) formulas to directly measured LDL-C in the Ghanaian population and to derive possible predictive equation for calculating LDL-C among our study population.

Lipoprotein concentrations and their distributions were analyzed in this study and we found significant differences between the mean values of F-LDL-C, N-LDL-C, A-LDL-C, and M-LDL-C with respect to D-LDL-C mean values. M-LDL-C values were significantly higher compared to the rest of the formulas except A-LDL-C (Table 1). This finding is not agreement with reports from a study conducted by Vujovic et al. in a Serbian population [11]. According to their work, mean LDL-C of participants directly measured was higher than those of calculated formulas; they reported a percentage difference of -6.9% for F-LDL-C and -3.9% of A-LDL-C [11]. We found a mean percentage differences of 12.39% and 23.54% for F-LDL-C and A-LDL-C, respectively (Table 2). In addition, Gupta and colleagues also found measured LDL-C to be higher than that obtained with the calculated formulas [23]. On the other side, Boshtam et al. found that mean levels of D-LDL-C were lower than F-LDL-C in Iranian population [24] which is consistent with results in this study. Our results showed that mean difference between the two methods was statistically significant (*P*<0.0001). In parallel, some studies have demonstrated similar trend with higher results with calculated LDL-C for F-LDL-C as compared to directly measured LDL-C [25, 26].

Reports from the original study for the development of Friedewald's formula provided a simple division of blood plasma TG by 5 for mg/dL or 2.2 for mmol/L [22]; however this formula does not provide accurate estimation of VLDL-C. In our course to evaluate the reliability of Friedewald's, Martin's, and Anandaraja's formula, we developed a new modified formula, which closely resembles Friedewald's that exhibited a simple division of patient's plasma TG by 4. Several studies have suggested alterative calculation in different populations which include TG/2.2, TG/2.5. TG/2.8, TG/3.0, TG/3.3, and TG/3.9 (mmol/L) [27, 28].

The difference between calculated LDL-C and directly measured LDL-C results can be important regarding risk classification for coronary heart disease among patients [29]. In 2008, a study done by Jun et al., among Koreans, showed that F-LDL-C was significantly different from D-LDL-C over the concentration ranges of both TC and TG. In the same study, the mean % ΔLDL was -9.1% and it was anticipated that this difference was critical for the evaluation of patients with hyperlipidemia [30]. In this study, mean values of F-LDL-C were lower than that of D-LDL-C in the first quartile of TC with a mean % ΔLDL-C of -5.0%. However, F-LDL-C values were higher compared to the directly measured LDL-C in the rest of the quartiles for TC and few ranges of TG and LDL-C (Table 3). Of note, LDL-C levels calculated with our modified formula (M-LDL-C) were statistically significantly higher compared to D-LDL-C in all ranges of TC and in most ranges of TC and that of LDL-C to correctly classify subjects at the clinical decision cut-off points in specific subgroups.

F-LDL-C had a sensitivity of 63.0% and specificity of 84.0% with negative predictive value of 87.0% and positive predictive 74.0% with a cut-off of 2.92 mmol/L in this study. M-LDL-C had a sensitivity of 0.60 and specificity of 0.88 with a cut-off value of 3.23mmol/L. These findings contrast with reports from a study by Martin et al. in South Africa. In their work, they recorded a higher sensitivity and specificity with a cut-off of 2.5 mmol/L [9]. The possible reason for these inconsistencies might be that F-LDL-c is poor in assessing direct LDL-C when the LDL-C values are high and the different study populations in the studies [31].

In general, there were strong correlations among the various formulas for estimating LDL-C concentrations. However, moderate correlations were observed between the directly measured LDL-C and the various methods. Among the three formulas used in this study, the Anandaraja's formula showed the least correlation with the directly measured LDL-C (Table 5). The observed correlations are lower compared with reports from previous studies [25, 32–34]. These studies reported correlations of 0.88 [25] and 0.86 [33] and 0.786 [32], respectively. Another study conducted among Japanese also found a positive correlation between F-LDL-C and D-LDL-C [34]. Furthermore, Anandaraja and colleagues also reported a Pearson's correlation coefficient of 0.97 between LDL-C measured by their formula and D-LDL-C which was better as compared to that for F-LDL-C [20]. Conversely, Kapoor et al. found a lower correlation between A-LDL-C and D-LDL-C which is in relative agreement with the lower correlation coefficient of 0.658 observed in this present study [35].

Bland-Altman graphs showed a clear relationship between both the directly measured LDL-C and the calculation formulas. The observed low bias can be well appreciated in all plots though the bias between N-LDL-C and D-LDL-C was the lowest. This indicates that the calculation formulas and the directly measured LDL-C methods are systematically producing similar results (Figure 2). Some previous studies have reported that Friedewald calculation demonstrates better agreement with directly measured LDL-C [23, 36].

This study has strength being the first study to compare different methods of estimating LDL-c concentration in Ghana and the West African subregion. However, our study is limited by the fact that both derived models must be further scrutinized and validated bearing in mind the differences in race and the specific character of the applied method of measurement.

## 5. Conclusion

The modified LDL-c (M-LDL-c) formula derived by this study yielded a better diagnostic accuracy compared to A-LDL-c and F-LDL-c equation and thus could serve as a substitute for D-LDL-c and F-LDL-c equation in the Ghanaian population. Taking into consideration the racial variances as well as the specific character of the applied method of measurement, the study findings underscore the need for scrutiny, validation, and reliability evaluations of the generated models, to ascertain their clinical use. Further work should also examine the performance of rick calculations by the various formulae.

## Figures and Tables

**Table 1:** Distribution of basic lipoprotein measurements.

	**TC**	**TG**	**HDL**	**Non-HDL**	**D-LDL-C**	**F-LDL-C**	**N-LDL-C**	**A-LDL-C**	**M-LDL-C**
	**mmol/L**	**mmol/L**	**mmol/L**	**mmol/L**	**mmol/L**	**mmol/L**	**mmol/L**	**mmol/L**	**mmol/L**
Mean	4.60	1.02	1.38	3.22	2.47	2.76**∗∗**	2.74**∗∗**	2.99**∗∗**	2.97**∗∗**
SD	1.20	0.57	0.36	1.13	0.71	1.05	1.04	1.02	1.08
1st Quartile	3.77	0.65	1.13	2.45	1.94	2.03	2.03	2.30	2.23
Median	4.45	0.88	1.33	3.09	2.43	2.64	2.63	2.87	2.64
3rd Quartile	5.21	1.23	1.59	3.78	2.90	3.28	3.26	3.54	3.28

**∗** **∗** ***p<0.001: significant in comparison to D-LDL-C*** *. TC: total cholesterol; TG: triglycerides; HDL-C: high-density lipoprotein cholesterol; LDL-C: low-density lipoprotein cholesterol; D-LDL-C: directly measured LDL-C; FLDL-C LDL-C calculated by Friedewald's formula; N-LDL-C, LDL-C calculated by Martin's formula; A-LDL-C, LDL-C calculated by Anandaraja's formula; M-LDL-C, LDL-C calculated by modified formula.*

**Table 2:** Mean percentage difference between D-LDL-C and calculated LDL-C.

	**∆ F-LDL-C (**%**)**	**∆ N-LDL-C (**%**)**	**∆A-LDL (**%**)**	**∆M-LDL-C (**%**)**
Mean	12.39	11.74	23.54	21.33
SD	27.34	29.25	30.45	28.43
1st Quartile	4.33	5.07	1.39	3.63
Median	13.79	13.51	23.84	23.8
3rd Quartile	29.63	28.55	44.81	39.11

SD: standard deviation; ΔA-LDL-C: mean percentage difference for Friedwald's formula; ΔA-LDL-C: mean percentage difference for Martin formula; ΔA-LDL-C: mean percentage difference for Anandaraja's formula; ΔA-LDL-C: mean percentage difference for modified formula. Mean percentage difference was calculated as [(calculated LDL-C)-(D-LDL-C)] *∕*D-LDL-C*∗*100].

**Table 3:** Means ± SDs and percentages of correctly classified subjects in risk categories regarding TC, TG and D-LDL-C concentrations given by NCEP ATP III.

							%** of subjects correctly classified by the following formulas**			
TC (mmol/L)	n	D-LDL-C	F-LDL-C	N-LDL-C	A-LDL-C	M-LDL-C	F	N	A	M
≤4.13	586	2.01±0.53	1.91±0.40**∗**	1.89±0.40**∗∗**	2.11±0.38**∗**	2.09±0.41**∗**	77.6%	77.9%	92.6%	86.4%
4.14-5.16	536	2.49±0.51	2.79±0.41**∗∗**	2.77±0.39**∗∗**	3.04±0.31**∗∗**	2.99±0.40**∗∗**	78.1%	77.9%	84.1%	78.9%
5.17-6.20	272	2.92±0.50	3.52±0.45**∗∗**	3.52±0.41**∗∗**	3.80±0.36**∗∗**	3.78±0.42**∗∗**	72.4%	76.4%	65.6%	64.6%
6.21-7.24	89	3.44±0.65	4.45±0.49**∗∗**	4.43±0.44**∗∗**	4.66±0.38**∗∗**	4.72±0.45**∗∗**	65.8%	70.3%	56.9%	48.1%
≥ 7.25	35	3.90±0.66	6.16±2.18**∗∗**	6.17±2.18**∗∗**	6.37±1.84**∗∗**	6.53±2.21**∗∗**	63.8%	68.9%	59.6%	50.8%
TG (mmol/L)										
≤1.13	1055	2.40±0.69	2.63±0.90**∗∗**	2.57±0.89**∗**	2.92±0.91	2.78±0.91**∗∗**	75.2%	79.1%	73.0%	80.6%
1.14-1.69	324	2.61±0.70	2.96±0.90**∗∗**	3.10±0.93**∗∗**	3.10±0.99	3.24±0.96**∗∗**	30.2%	32.4%	29.3%	29.6%
1.70-2.25	76	2.75±0.69	3.18±1.20	3.30±0.89*∗*	3.29±1.20	3.58±1.19**∗**	19.7%	23.7%	25.0%	22.4%
2.26-2.82	40	2.68±0.75	3.49±2.82	3.74±2.74	3.51±2.49	4.01±2.82	11.4%	5.0%	2.5%	10.0%
2.83-4.52	23	2.74±1.01	3.00±1.05	3.43±0.96	3.03±1.02	3.75±1.07	4.3%	8.7%	8.7%	17.4%
LDL-C (mmol/L										
≤2.59	903	2.01±0.38	2.33±0.65**∗∗**	2.31±0.65**∗∗**	2.61±0.68	2.53±0.67**∗∗**	80.0%	80.4%	80.0%	81.4%
2.60-3.35	447	2.92±0.22	3.05±0.90	3.04±0.89	3.23±0.88	3.27±0.92**∗∗**	41.4%	42.9%	31.7%	34.1%
3.36-4.12	142	3.62±0.18	3.98±0.91**∗**	3.95±0.92**∗**	4.18±0.93	4.22±0.97	31.9%	31.4%	19.2%	22.6%
4.13-4.89	21	4.36±0.21	5.68±2.54	5.64±2.57	5.72±2.26	5.94±2.26**∗**	8.2%	9.5%	9.8%	42.5%
≥4.90	5	5.54±0.44	6.08±2.12	6.06±2.69	6.07±2.69	6.40±2.06	10.6%	8.8%	3.5%	7.7%

**∗** ***p<0.05,*** **∗** **∗** ***p<0.0001: significant compared to D-LDL-C.***

*TC: total cholesterol; TG: triglycerides; HDL-C: high-density lipoprotein cholesterol; LDL-C: low-density lipoprotein cholesterol; D-LDL-C: directly measured LDL-C; FLDL-C, LDL-C calculated by Friedewald's formula; N-LDL-C, LDL-C calculated by Martin's formula; A-LDL-C, LDL-C calculated by Anandaraja's formula; M-LDL-C, LDL-C calculated by modified formula.*

**Table 4:** Diagnosis performances of the various formulas.

**Formulas**	**Cut-off point**	**AUC**	**95**%** CI**	**Sensitivity (95**%** CI)**	**Specificity (95**%**CI)**	**NPV**	**PPV**
F-LDL-C	2.92	0.80	0.78-.83	0.63(0.60-0.67)	0.84(0.82-0.87)	0.87	0.74
N-LDL-C	2.92	0.81	0.78-.83	0.63(0.60-0.66)	0.85(0.82-0.87)	0.87	0.78
A-LDL-C	2.96	0.77	.75-.78	0.68(0.65-0.72)	0.73(0.70-0.77)	0.68	0.73
M-LDL-C	3.23	0.81	.78-.83	0.60(0.56-0.64)	0.88(0.85-0.90)	0.72	0.72

AUC: area under curve; CI: confidence interval; NPV: negative predictive value; PPV: positive predictive value; FLDL-C, LDL-C calculated by Friedewald's formula; N-LDL-C, LDL-C calculated by Martin's formula; A-LDL-C, LDL-C calculated by Anandaraja's formula; M-LDL-C, LDL-C calculated by modified formula.

**Table 5:** Pearson correlation coefficient (r) (bold) and coefficient of determination (R^2^) (italics) between formulas.

Formulas	F-LDL	N-LDL	A-LDL	M-LDL	D-LDL-C
F-LDL-C (mmol/L)		**0.995**	**0.947**	**0.994**	**0.693**
		**<0.0001**	**<0.0001**	**<0.0001**	**<0.0001**
N-LDL-C (mmol/L)	*0.991*		**0.937**	**0.999**	**0.693**
	*<0.0001*		**<0.0001**	**<0.0001**	**<0.0001**
A-LDL-C (mmol/L)	*0.898*	*0.878*		**0.935**	**0.658**
	*<0.0001*	*<0.0001*		**<0.0001**	**<0.0001**
M-LDL-C (mmol/L)	*0.989*	*0.999*	*0.8743*		**0.693**
	*<0.0001*	*<0.0001*	*<0.0001*		**<0.0001**
D-LDL-C (mmol/L)	*0.481*	*0.480*	*0.434*	*0.481*	
	*<0.0001*	*<0.0001*	*<0.0001*	*<0.0001*	

FLDL-C, LDL-C calculated by Friedewald's formula; N-LDL-C, LDL-C calculated by Martin's formula; A-LDL-C, LDL-C calculated by Anandaraja's formula; M-LDL-C, LDL-C calculated by modified formula.

## Data Availability

The data used to support the findings of this study are included within the article.
